# Serum Immunoglobulin G4 and Immunoglobulin G1 for Distinguishing Immunoglobulin G4-Associated Cholangitis From Primary Sclerosing Cholangitis

**DOI:** 10.1002/hep.26977

**Published:** 2014-04-01

**Authors:** Kirsten Boonstra, Emma L Culver, Lucas Maillette de Buy Wenniger, Marianne J van Heerde, Karel J van Erpecum, Alexander C Poen, Karin MJ van Nieuwkerk, BW Marcel Spanier, Ben JM Witteman, Hans ARE Tuynman, Nan van Geloven, Henk van Buuren, Roger W Chapman, Eleanor Barnes, Ulrich Beuers, Cyriel Y Ponsioen

**Affiliations:** 1 Department of Gastroenterology and Hepatology, Academic Medical CenterAmsterdam, the Netherlands; 2 Translational Gastroenterology Unit, John Radcliffe HospitalOxford, United Kingdom; 3 Oxford NIHR Biomedical Research Center, and NDM, University of OxfordOxford, United Kingdom; 4 Department of Gastroenterology and Hepatology, Erasmus University Medical CenterRotterdam, the Netherlands; 5 Department of Gastroenterology and Hepatology, University Medical Center UtrechtUtrecht, the Netherlands; 6 Department of Gastroenterology and Hepatology, Isala ClinicsZwolle, the Netherlands; 7 Department of Gastroenterology and Hepatology, VU Medical CenterAmsterdam, the Netherlands; 8 Department of Gastroenterology and Hepatology, Rijnstate HospitalArnhem, the Netherlands; 9 Department of Gastroenterology and Hepatology, Gelderse Vallei HospitalEde, the Netherlands; 10 Department of Gastroenterology and Hepatology, Medical Center AlkmaarAlkmaar, the Netherlands; 11 Clinical Research Unit, Academic Medical CenterAmsterdam, the Netherlands

## Abstract

The recent addition of immunoglobulin (Ig)G4-associated cholangitis (IAC), also called IgG4-related sclerosing cholangitis (IRSC), to the spectrum of chronic cholangiopathies has created the clinical need for reliable methods to discriminate between IAC and the more common cholestatic entities, primary (PSC) and secondary sclerosing cholangitis. The current American Association for the Study of Liver Diseases practice guidelines for PSC advise on the measurement of specific Ig (sIg)G4 in PSC patients, but interpretation of elevated sIgG4 levels remains unclear. We aimed to provide an algorithm to distinguish IAC from PSC using sIgG analyses. We measured total IgG and IgG subclasses in serum samples of IAC (n = 73) and PSC (n = 310) patients, as well as in serum samples of disease controls (primary biliary cirrhosis; n = 22). sIgG4 levels were elevated above the upper limit of normal (ULN = >1.4 g/L) in 45 PSC patients (15%; 95% confidence interval [CI]: 11-19). The highest specificity and positive predictive value (PPV; 100%) for IAC were reached when applying the 4× ULN (sIgG4 > 5.6 g/L) cutoff with a sensitivity of 42% (95% CI: 31-55). However, in patients with a sIgG4 between 1× and 2× ULN (n = 38/45), the PPV of sIgG4 for IAC was only 28%. In this subgroup, the sIgG4/sIgG1 ratio cutoff of 0.24 yielded a sensitivity of 80% (95% CI: 51-95), a specificity of 74% (95% CI: 57-86), a PPV of 55% (95% CI: 33-75), and a negative predictive value of 90% (95% CI: 73-97). *Conclusion*: Elevated sIgG4 (>1.4 g/L) occurred in 15% of patients with PSC. In patients with a sIgG4 >1.4 and <2.8 g/L, incorporating the IgG4/IgG1 ratio with a cutoff at 0.24 in the diagnostic algorithm significantly improved PPV and specificity. We propose a new diagnostic algorithm based on IgG4/IgG1 ratio that may be used in clinical practice to distinguish PSC from IAC. (Hepatology 2014;59:1954–1963)

Primary sclerosing cholangitis (PSC) represents the most common chronic immune-mediated cholangiopathy among men, but is still rare given its prevalence, ranging from 0 to 16.2 per 100,000 inhabitants.^1^ Differentiation of PSC from other chronic cholangiopathies can be extremely challenging.^2^ However, a correct diagnosis of PSC is crucial to optimize the surveillance of disease progression, because chronic inflammation of the intra- and extrahepatic bile ducts (BDs) may ultimately lead to cirrhosis and liver failure, with median survival rates until death or liver transplantation (LT) from 12 to 21 years.^3^ PSC is more common in men than in women (2:1) and can occur at any age, with a peak incidence around 40.^3^ PSC is strongly associated with inflammatory bowel diseases (IBDs), often classified as ulcerative colitis, and patients have a poor prognosis as a result of liver failure and an increased risk for developing colorectal and biliary malignancies.^4^ The only known curative therapy available to date is orthotopic liver transplantation, although the use of ursodeoxycholic acid (UDCA) may improve surrogate markers of disease progression, particularly in early-stage patients, and is still widely prescribed at moderate doses in nonfibrotic patients.^5^

Discerning PSC from other chronic cholangiopathies has, in recent years, become even more challenging, with the establishment of a new disease entity, immunoglobulin (Ig)G4-associated cholangitis (IAC), or IgG4-related sclerosing cholangitis (IRSC),^6^ with which PSC shares male predominance, a cholestatic serum enzyme pattern, and cholangiographic features with intra- and/or extrahepatic BD strictures.^7^–^9^ IAC represents the biliary manifestation of a fully separate systemic disease entity: IgG4-related disease (IgG4-RD). IgG4-RD includes various organ manifestations, among which the pancreas and biliary tree appear most frequently affected. IgG4-RD is often associated with elevated IgG4 serum levels^10^ and is characterized by IgG4-positive plasmacellular tissue infiltrates.^11^ In striking contrast to PSC, IAC fully responds to corticosteroid treatment when diagnosed in time.^10^

IgG4 is one of the four known subtypes of IgG molecules. Apart from being the least abundant IgG in healthy people (typically forming <5% of total serum IgG), IgG4 has unique biochemical properties, of which the ability to exchange its half molecules, thus yielding antibodies (Abs) with dual antigen affinities, is the most intriguing.^12^ Unusually high IgG4 serum levels were first reported in patients with autoimmune pancreatitis (AIP) and have since been associated also with other organ manifestations of IgG4-RD, including IAC.^13^ A gold standard for diagnosing AIP and IAC is still lacking. Therefore, use of one of the currently accepted sets of diagnostic criteria has become common practice, of which the HISORt criteria for diagnosing AIP arguably are the most widely applied^14^ and which were adapted for use in suspected IAC patients.^7^,^15^,^16^

Because IgG4-RD can be adequately controlled in the vast majority of cases by immunosuppressive medication, it is crucial to discriminate IAC cases from PSC patients.^17^ IAC becomes symptomatic, on average, in men of an older age (60-80 years). Upon careful history taking, IAC patients often have other organ manifestations of IgG4-RD, when compared to those with PSC. An elevated IgG4 serum level is still the pivotal finding that leads to the diagnosis, and elevated IgG4 levels are thus firmly anchored in the current diagnostic criteria of IAC. The American Association for the Study of Liver Diseases practice guideline on the diagnosis and management of PSC published recently in this journal suggested measuring specific Ig (sIg)G4 in all patients with possible PSC, to exclude IAC.^18^ However, elevated levels of IgG4 have also been shown in 9%-27% of clear-cut PSC patients.^19^–^23^ Therefore, it is currently unclear how to interpret an elevated sIgG4 level in a patient with alleged PSC. Furthermore, no data exist currently regarding levels of other IgG subclasses in these patients. Given that specific IgG4-positive plasma/B-cell clones seem to play a central role in the pathogenesis of IAC,^24^ we hypothesized that IgG4 may be specifically increased in IAC, whereas a nonspecific increase in IgG of different IgG subclasses could be expected in other immune-mediated disorders, such as PSC. Therefore, in search of a reliable algorithm, we determined total IgG and IgG subclasses in serum samples of two large independent PSC and IAC cohorts as well as in serum samples of a disease control group with primary biliary cirrhosis (PBC).

## Patients and Methods

### Study Subjects

The Dutch (the Netherlands; NL) cohort consisted of 132 PSC and 27 IAC patients. In addition, 22 PBC patients were included as liver disease controls. PSC and PBC patients were randomly selected from a large population-based study in the Netherlands (Epi PSC PBC study) containing 695 PSC patients and 1,035 PBC cases included between March 2008 and December 2011. Between August 2004 and July 2012, IAC patients were referred to two large tertiary referral centers in the Netherlands: the Academic Medical Center in Amsterdam and the Erasmus University Medical Center in Rotterdam. The UK cohort consisted of 178 PSC and 46 IAC patients who were enrolled in a prospective database in the Oxford Radcliffe Hospitals from August 2010 onward. PSC patients were selected from a larger database of 346 patients attending the John Radcliffe Hospital in Oxford from between 2001 and 2012. PSC patients from the UK cohort were entered consecutively into a prospective PSC database at diagnosis or on referral to the center. Patients were included in this study if full clinical details, biliary imaging, and histology (where available) were reviewed and they had been followed up in the hepatology clinic within the last 3 years. Serum samples were collected from 2005 onward.

All medical records were thoroughly reviewed onsite for confirmation of diagnosis and data retrieval. Patients with a PSC- or PBC-autoimmune hepatitis overlap syndrome and patients using corticosteroids and/or thiopurines during 6 months preceding serum sample collection were excluded. The study was approved by the central committee for research ethics in Utrecht and all participating local ethics committees in the Netherlands (http://www.trialregister.nl: NTR2813) and by the Oxford Research Ethics Committee (RECA 10/H0604/51) funded by a Wellcome Trust Fellowship grant (095160/Z/10/Z).

### Case Ascertainment

**Fig. 1:**
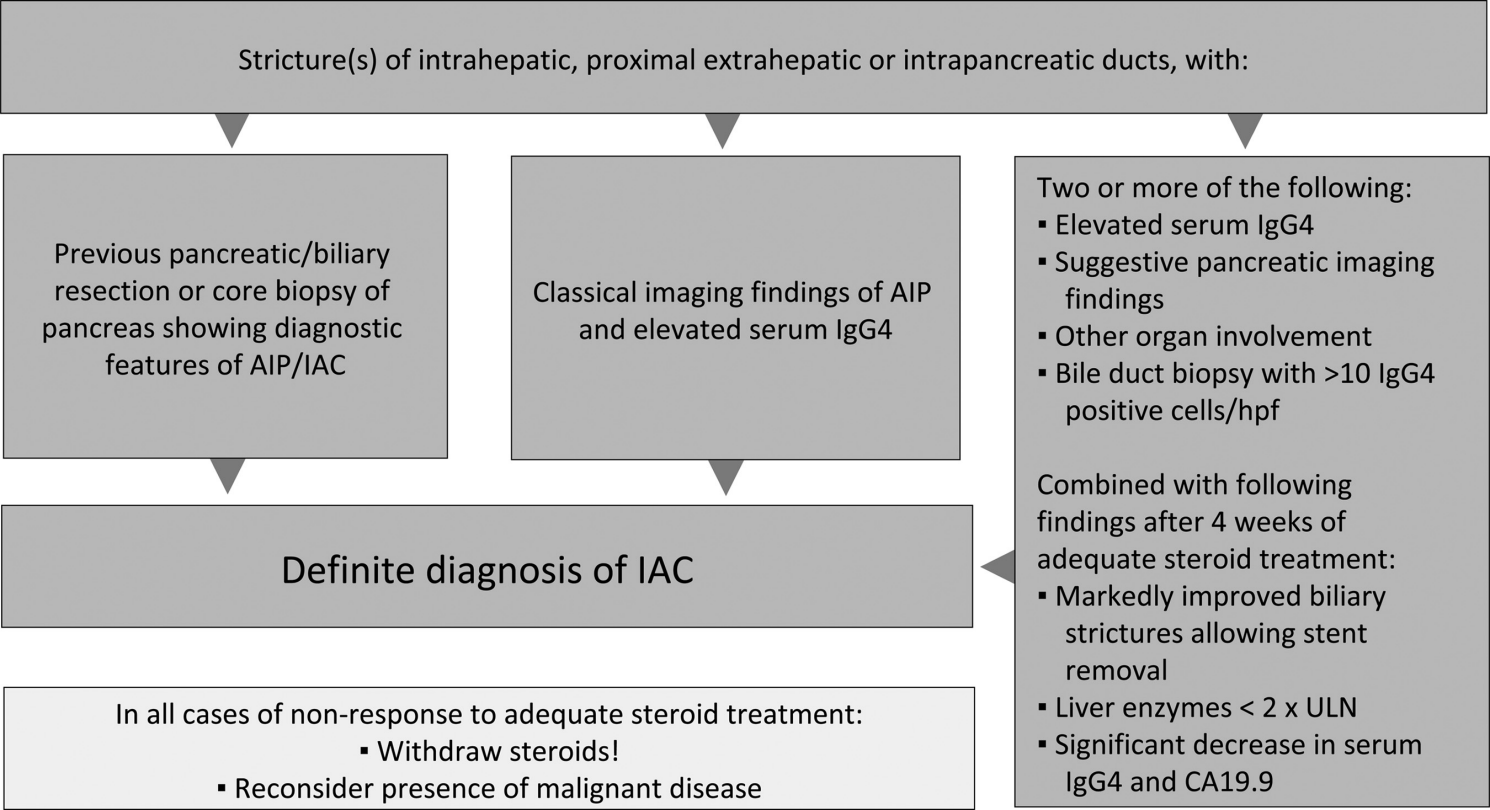
HISORt diagnostic criteria (*h*istology, *i*maging, *s*erology, other *o*rgan involvement, and *r*esponse to *t*herapy) for IgG4-associated cholangitis (IAC). Adapted from Ghazale et al.,^7^ Alderlieste et al.,^15^ and Maillette de Buy Wenniger et al.^16^

Diagnosis of PSC was based on (1) clinical presentation, that is, pruritus, pain in the right upper abdominal quadrant, fatigue, weight loss, or episodes of fever and/or (2) elevated alkaline phosphatase (ALP) and gamma-glutamyltransferase that was otherwise unexplained, (3) presence of characteristic BD changes with multifocal strictures and segmental dilatations on endoscopic retrograde cholangiography (ERC) or magnetic resonance cholangiography (MRC), (4) liver histology, and (5) no evidence for secondary sclerosing cholangitis (SSC). Criteria 2 and 3 were considered mandatory for PSC, whereas criterion 4 confirmed the diagnosis where available. No PSC patient was allowed to have pancreatic imaging suggestive of AIP or other organ involvement. The HISORt criteria (histology, imaging, serology, other organ involvement, and response to therapy; Fig. 1) were applied for diagnosing IAC. Diagnosis of PBC was based on a combination of (1) clinical presentation and/or (2) elevation of ALP of liver origin for at least 6 months, (3) presence of antimitochondrial Abs (≥1:40) in serum, and (4) histological features of florid BD lesions. Criteria 2 and 3 were considered mandatory for PBC, whereas criterion 4 confirmed the diagnosis where available.^5^

### Serology

Serum total IgG and subclasses IgG1, IgG2, IgG3, and IgG4 were measured using automated nephelometry (BN ProsPec Siemens in the Netherlands and BNII Siemens in Oxford). Samples were monitored for antigen excess by two separate strategies. First, IgG4 levels plus IgG1 levels for each patient were correlated to total IgG levels to check for >15% discordance; this gives an indication of antigen excess. Samples from patients with a normal IgG4 level were checked at multiple dilutions to check for nonlinearity and probable antigen excess.

### Statistical Analysis

Mann-Whitney's U test was performed for comparing continuous data without a normal distribution, and *t* tests were used to compare normally distributed continuous data. The chi-square test or Fisher's exact test were used for comparing categorical data. The one-way analysis of variance test and Kruskal-Wallis' test were used for comparing continuous data between three groups. Receiver operator characteristic (ROC) curves were plotted to determine optimal cut-off values for sIgG4 and for subclass ratio levels for distinguishing IAC from PSC. The optimal cut-off value was defined as the cutoff corresponding to the point on the ROC curve closest to the sens = 1 spec = 1 optimum. Diagnostic algorithms were compared using McNemar's test with regard to sensitivities and specificities and with the generalized score statistic, as proposed by Leisenring with regard to positive (PPV) and negative predictive value (NPV).^25^ Statistical analyses were performed using SPSS v. 19·0 software (SPSS, Inc., Chicago, IL) and R (package *DTComPair*).^26^ *P* < 0.05 was considered statistically significant.

## Results

### Elevated Serum IgG4 (>1.4 g/L) Occurs in 15% of PSC Patients

In total, serum IgG and IgG subclasses were measured in 310 PSC, 73 IAC, and 22 PBC patients (demographics are shown in Table 1). PSC patients were diagnosed at a mean age of 44.0 (standard deviation [SD]: 16.2) and IAC patients at a mean age of 62.5 years (SD, 14.1; P < 0.001; *t* test). Elevated sIgG4 levels (>1.4 g/L) were observed in 45 PSC patients (15%; 95% CI: 11-19; Fig. 2). Seven (2%) had a sIgG4 greater than 2× upper limit of normal (ULN). None of the PSC patients had a sIgG4 greater than 4× ULN. Notably, 7 (10%) IAC patients had a sIgG4 <1.4 g/L.

**Table 1:** Demographics and Serum Total IgG and IgG Subclasses of PSC and IAC Patients and PBC controls

Variable	PSC	IAC	*P* Value	PBC
No.	310	73		22
Male, n (%)	170 (55)	62 (85)	<0.001	0 (0)
Age at diagnosis, years (mean [SD])	44.0 (16.2)	62.5 (14.1)	<0.001	49.5 (11.5)
Age at blood sampling, years (mean [SD])	51.2 (16.5)	64.1 (12.9)	<0.001	60.0 (9.5)
IBD, n (%)	180 (58)	6 (8)	<0.001	0 (0)
Total IgG, g/L (mean [SD])	13.5 (4.1)	17.0 (8.3)	0.001	13.5 (4.7)
IgG1, g/L (mean [SD])	9.1 (3.5)	9.6 (5.0)	0.490	9.1 (3.2)
IgG2, g/L (mean [SD])	3.5 (1.6)	4.5 (2.1)*	<0.001	4.0 (1.9)
IgG3, g/L (median [IQR])	0.4 (0.3-0.6)	0.4 (0.3-0.7)	0.288	1.2 (0.7-1.6)
IgG4, g/L (median [IQR])	0.5 (0.3-1.0)	4.6 (2.2-11.1)	<0.001	0.3 (0.2-0.5)
IgG4 >1.40 g/L, n (%)	45 (15)	66 (90)	<0.001	0 (0)
IgG4 >2.80 g/L, n (%)	7 (2)	51 (70)	<0.001	0 (0)
IgG4 >5.60 g/L, n (%)	0 (0)	31 (42)	<0.001	0 (0)

* Based on 64 IAC patients.

**Fig. 2:**
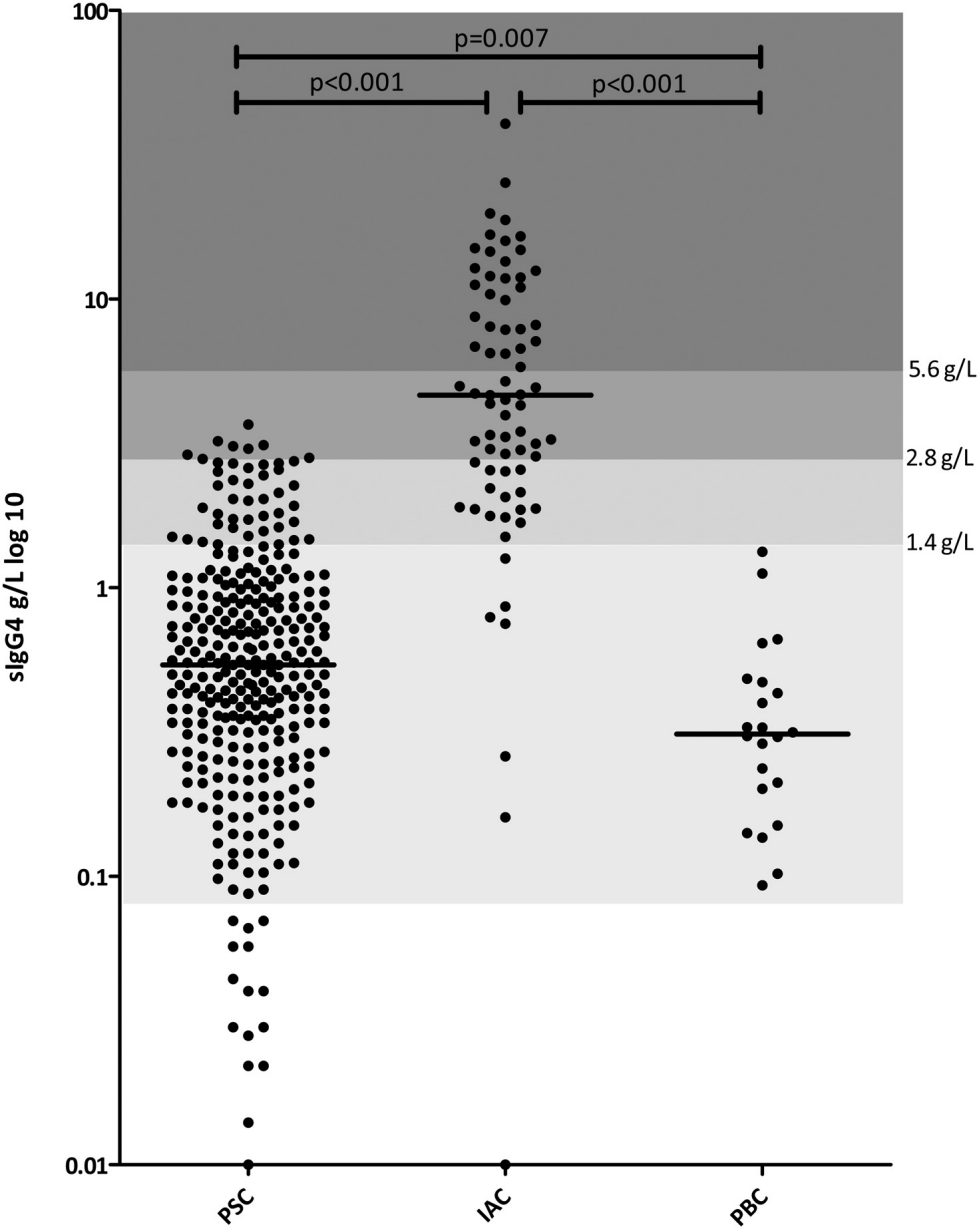
Scatterplot of sIgG4 in PSC, IAC, and PBC patients. Lower limit of normal (LLN)−1×, 1×-2×, 2×-4x, and >4x ULN are marked with different shades of gray. The bar across each column represents the median value. Statistic bars represent PSC versus PBC, PSC versus IAC, and IAC versus PBC comparisons by Mann-Whitney's U test.

When comparing PSC patients with an elevated sIgG4 to patients with normal sIgG4 levels, mean serum albumin levels were lower in patients with a sIgG4 >1.4 g/L (42 [SD, 5] vs. 44 g/L [SD, 4]; *P* = 0.012, *t* test). Median serum bilirubin levels (13 [interquartile range [IQR]: 8-21] vs. 11 µmol/L [IQR, 7-16]; *P* = 0.212, Mann-Whitney's U test) and ALP levels (323 [IQR, 175-578] vs. 290 U/L [IQR, 176-485]; *P* = 0.414, Mann-Whitney's U test) were not significantly different. Mean age at PSC diagnosis of patients with an elevated sIgG4 did not differ from patients with a normal sIgG4 <1.4 g/L (45.3 [SD, 18.0] vs. 43.7 years [SD, 15.8]; *P* = 0.551, *t* test). Median time between PSC diagnosis and blood sampling was similar between groups (49 [IQR, 2-114] vs. 71 months [32-131]; *P* = 0.116, Mann-Whitney's U test).

Mean age at diagnosis was significantly different form IAC patients (45.3 [SD, 18.0] vs. 63.1 years [SD, 13.5]; *P* < 0.001, *t* test). All PSC patients with an elevated sIgG4 were scrutinized for signs of IAC. None of the PSC patients with an elevated sIgG4 had clinical signs or organ manifestations of IgG4-RD. Twenty-nine of forty-five (64%) patients with PSC and an elevated sIgG4 had liver biopsies; of these, 16 had tissue staining for IgG4 monoclonal Ab (Supporting Table 1). Tissue IgG4 was >10/HPF (per high-power field) in 3 of 16 liver biopsies (median, 12; mean, 20; range, 12-35 IgG4/3 HPF). All 3 patients had a sIgG4 between 1.4 and 2.8g/L. Tissue IgG4 was <10/HPF in 13 liver biopsies, of which 9 had a serum IgG4 between 1.4 and 2.8g/L and 3 had a serum IgG4 >2.8g/L. None of the 16 biopsies showed histological characteristics of IAC. Conversely, IAC patients with a sIgG4 <1.4 g/L were reviewed for a possible diagnosis of PSC. None had concomitant IBD, 6 of 7 had pancreatic disease and responded to corticosteroid treatment, and 1 of 7 with isolated IAC had classical histology and abundant IgG4 plasma cell staining in a liver resection specimen.

The majority of IAC patients had pancreatic involvement. Eleven of forty-seven (23.4%) patients only in the Oxford cohort had IAC without pancreatic involvement; 8 of these 11 had other radiological and histologically confirmed organ involvement, including lung (2), renal (1), sialoadenitis (1), mesenteric fibrosclerosis (2), retroperitoneal fibrosis (1), and colonic (3) involvement. In the case of the colon, a lymphoplasmacytic infiltrate with abundant IgG4 cells (>50/HPF) was observed with no polypoid lesions, storiform fibrosis, or phlebitis. Three of forty-seven (6.4%) patients had isolated IAC; 2 had resections to exclude cholangiocarcinoma with classical histology (lymphoplasmacytic infiltrate, storiform fibrosis, and phlebitis) and IgG4-positive plasma cell counts of greater than 50/HPF, and 1 had an elevated serum IgG4 >4× ULN as well as clinical, biochemical, and radiological response with stricture resolution after 3 months of corticosteroids with no tissue diagnosis. Likewise, in the Dutch cohort, the majority (88.9%) had pancreatic involvement at any point during follow-up; several cases also showed involvement of other organs, such as the salivary glands. Only 3 of 27 (11.1%) patients had isolated biliary IgG4-RD; none of these had other radiological or histologically confirmed organ involvements, but all had elevated serum IgG4 and elevated numbers of IgG4-positive cells in their liver tissue, cholestasis, and alterations on ERC or MRC suggestive of sclerosing cholangitis. All 3 showed a good clinical response to immunosuppressive treatment.

When comparing the other sIgG subclass levels between PSC and IAC patients, IgG1 and IgG3 levels were similar and mean sIgG2 levels were higher in IAC patients than in PSC patients (4.5 vs. 3.5; *P* < 0.001, *t* test; Table 1).

### The sIgG4 ULN Cutoff Is Insufficient for Distinguishing IAC From PSC

**Fig. 3:**
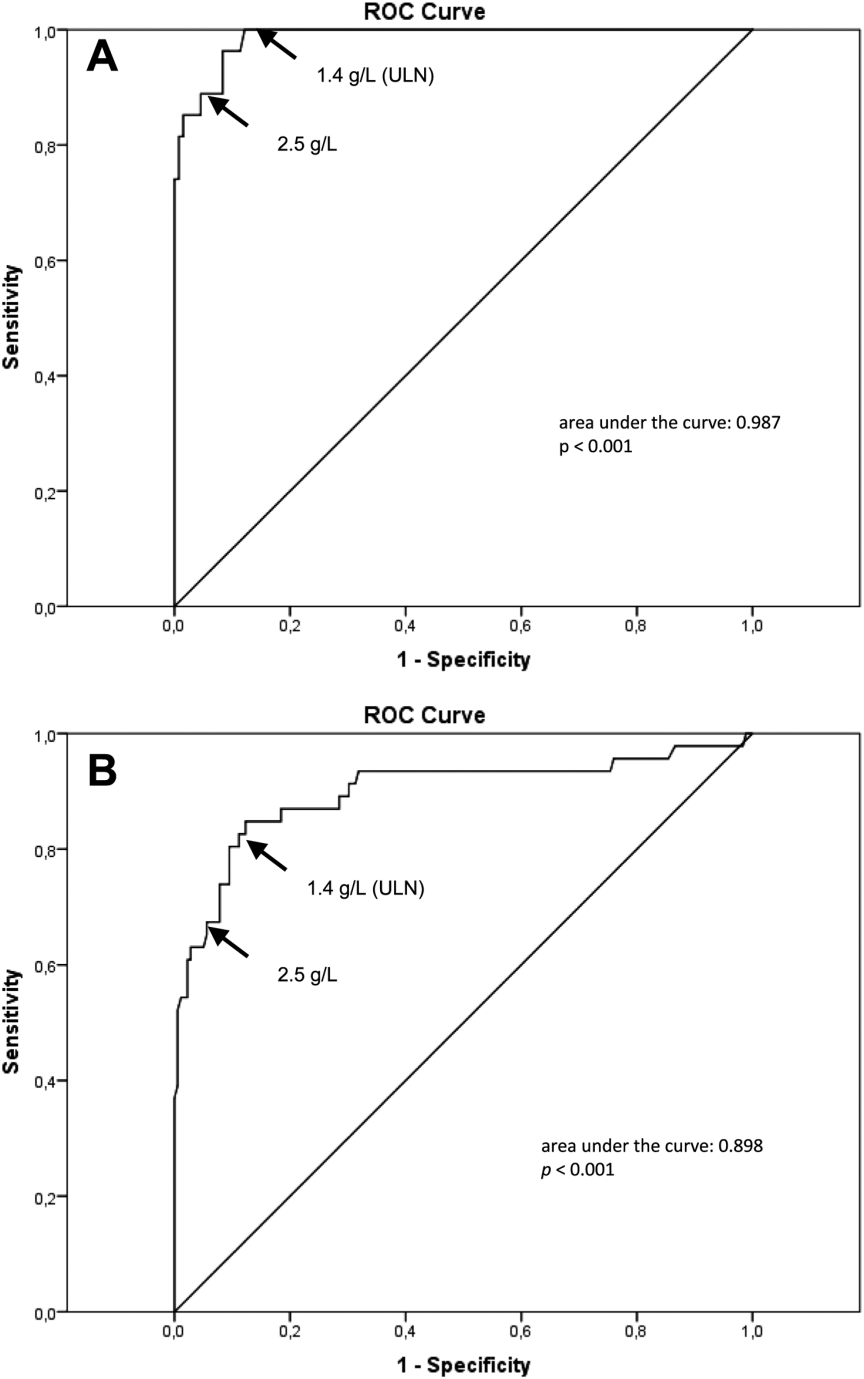
ROC curves of sIgG4 for diagnosis of IAC versus PSC patients in the test (A) and validation cohorts (B). Arrows are pointing toward the ULN of sIgG4 (1.4 g/L) and the optimal cut-off value (2.5 g/L).

The ULN cutoff for sIgG4 (1.4 g/L) yields a sensitivity of 90% (95% confidence interval [CI]: 81-96) with a specificity of 85% (95% CI: 81-89) for IAC (Table 2). PPV was only 59% (95% CI: 50-69), whereas NPV was 97% (95% CI: 95-99). The vast majority (38 of 45) of PSC patients with a sIgG4 level >1.4 g/L fell in the >1.4 to <2.8 range, where PPV for IAC was only 28%. Increasing the cut-off level to 2× ULN decreased the sensitivity of sIgG4 to 70% (95% CI: 58-80), whereas specificity and PPV increased to 98% (95% CI: 95-99) and 88% (95% CI: 76-95), respectively, and NPV was only slightly reduced from 97% to 93% (95% CI: 90-96). The highest specificity and PPV (100%) for IAC were reached when applying the 4× ULN cutoff with a sensitivity of 42% (95% CI: 31-55). In search of the optimal sIgG4 cut-off value for distinguishing IAC from PSC, we performed ROC analyses (Fig. 3). We first determined the optimal cut-off value in the Dutch cohort, serving as a test cohort. Second, we tested the performance of this new cut-off value in the UK patients, serving as a validation cohort. The ROC curve of the test cohort showed that a sIgG4 cutoff at 2.5 g/L yielded the optimal combination of sensitivity and specificity (Table 2). However, 7 (5%) PSC patients in the test cohort and 9 (5%) PSC patients in the validation cohort had a sIgG4 >2.5 g/L, and 3 (11%) IAC patients in the test cohort and 15 (33%) IAC patients in the validation cohort had a sIgG4 <2.5 g/L. These results illustrate the limited diagnostic value provided by moderately elevated sIgG4 when attempting to distinguish IAC from PSC.

**Table 2:** Performance of Serum IgG4 in Distinguishing IAC from PSC

	IgG4 >1.4 g/L	IgG4 >2.8 g/L	IgG4 >5.6 g/L	IgG4 >2.5 g/L Test (NL) Cohort	IgG4 >2.5 g/L Validation (UK) Cohort	New Algorithm (Fig. 5)
Sensitivity, % (95% CI)	90 (81-96)	70 (58-80)	42 (31-55)	89 (70-97)	67 (52-80)	86 (76-93)
Specificity, % (95% CI)	85 (81-89)	98 (95-99)	100 (98-100)	95 (89-98)	95 (90-98)	95 (91-97)
PPV, % (95% CI)	59 (50-69)	88 (76-95)	100 (86-100)	77 (58-90)	78 (61-89)	79 (68-87)
NPV, % (95% CI)	97 (95-99)	93 (90-96)	88 (84-91)	98 (93-99)	92 (87-95)	97 (94-98)

### Serum IgG4/IgG1 Ratio Is Helpful in Distinguishing IAC From PSC When sIgG4 Is Moderately Elevated (>1.4 to <2.8 g/L)

Forty-five PSC patients (15%; 95% CI: 11-19) had a sIgG4 above 1.4 g/L (Fig. 2) and would erroneously be categorized as IAC patients. To determine whether sIgG subclasses other than sIgG4 may help to reliably distinguish PSC from IAC in patients with an elevated sIgG4, we compared sIgG subclass levels between PSC patients with and without an elevated sIgG4 and compared subclass levels and sIgG4/subclass ratios between PSC and IAC patients with a sIgG4 >1.4 g/L. In PSC patients with a sIgG4 >1.4 g/L, mean sIgG1 (ULN, 11.4 g/L) and sIgG2 (ULN, 6.4 g/L) levels were higher than in PSC patients with a sIgG4 <1.4 g/L (10.7 [SD, 3.6] vs. 8.9 [SD, 3.4]; *P* = 0.001 and 4.4 [SD, 1.8] vs. 3.3 [SD, 1.5]; *P* < 0.001, respectively). Median sIgG3 (ULN, 1.1 g/L) levels were not significantly different (0.5 [IQR, 0.3-0.7] vs. 0.4 [IQR, 0.3-0.6]; *P* = 0.103). When comparing PSC and IAC patients with a sIgG4 >1.4 g/L, sIgG1, sIgG2, and sIgG3 levels were not significantly different (data not shown). All sIgG4/subclass ratios were higher in IAC patients than in PSC patients (Table 3). To determine the most reliable subclass ratio for distinguishing IAC from PSC, we used ROC curves. The sIgG4/sIgG1 ratio showed the largest area under the ROC curve (AUROC) and reached the optimal combination of sensitivity and specificity at 0.24 (Table 3; and Supporting Fig. 1). In patients with a sIgG4 >1.4 g/L, the sIgG4/sIgG1 ratio cut-off value of 0.24 yielded a sensitivity of 92% (95% CI: 82-97), specificity of 64% (95% CI: 49-78), a PPV of 79% (95% CI: 68-87), and an NPV of 85% (95% CI: 68-94). In patients with a sIgG4 >2.8 g/L, the sIgG4/sIgG1 ratio was not of additional value in distinguishing IAC from PSC. However, in patients with a moderately elevated sIgG4 between 1× and 2× ULN, the ratio cutoff of 0.24 was of discriminating value, with a sensitivity of 80% (95% CI: 51-95), a specificity of 74% (95% CI: 57-86), a PPV that improved from 28% to 55% (95% CI: 33-75), and an NPV of 90% (95% CI: 73-97; Fig. 4). A proposed algorithm for distinguishing IAC from PSC in all patients based on sIgG4 and sIgG1 levels is shown in Fig. 5.

**Table 3:** Serum IgG4/Subclass Ratio Analysis

Subclass Ratio	PSC	IAC	*P* Value	AUROC (95% CI)
IgG4/IgG1, median (IQR)	0.21 (0.15-0.28)	0.61 (0.32-1.17)	<0.001	0.887 (0.828-0.946)
IgG4/IgG2, median (IQR)	0.51 (0.41-0.51)	1.10 (0.68-2.13)	<0.001	0.859 (0.788-0.931)
IgG4/IgG3, median (IQR)	4.46 (2.79-6.74)	10.14 (6.64-20.82)	<0.001	0.832 (0.752-0.913)

**Fig. 4:**
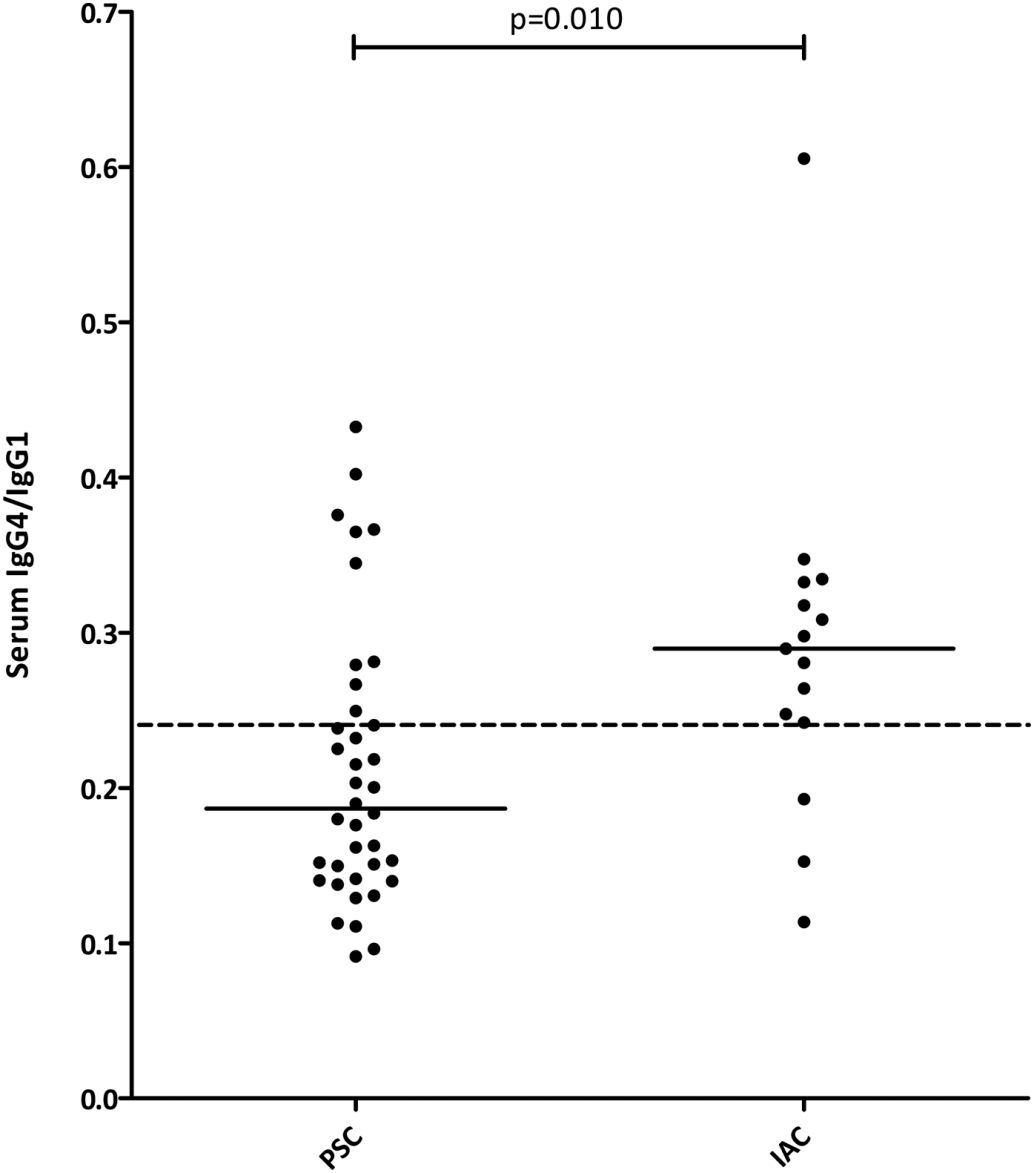
Scatterplot of sIgG4/sIgG1 in PSC and IAC patients with a sIgG4 between 1.4 and 2.8 g/L. The ratio cut-off value of 0.24 is shown as a dotted line. The bar across each column represents the median value.

**Fig. 5:**
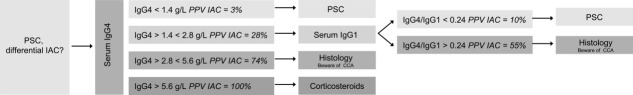
Proposed algorithm for distinguishing IAC from PSC using sIgG4 and sIgG4/sIgG1 ratio analysis.

### Applying a Diagnostic Algorithm Instead of the ULN as a Diagnostic Tool Is Helpful in Distinguishing IAC From PSC

When using the ULN cutoff (1.4 g/L), 45 PSC patients (15%) would erroneously be diagnosed with IAC. When applying the algorithm shown in Fig. 5, 28 (62%) of these 45 PSC patients would not be classified as IAC on the basis of sIgG profiles alone. In the remaining 17 (38%) patients, the advice would be to look for characteristic histology of IAC. None of the PSC patients would be classified as having IAC instead of PSC solely based on sIgG4 and corticosteroid treatment would only be started in patients with a sIgG4 above 4× ULN or patients with histological signs of IgG4-RD. When considering all sIgG4 values above 2× ULN or sIgG4 values between 1× and 2× ULN, together with a sIgG4/sIgG1 ratio above 0.24, as positive test results, the proposed algorithm yielded a sensitivity of 86% (95% CI: 76-93), a specificity of 95% (95% CI: 91-97), a PPV of 79% (95% CI: 68-87), and an NPV of 97% (95% CI: 94-98; Table 2, last column). Compared to the sole use of the ULN (Table 2, first column), this entailed a significant improvement in specificity and PPV (*P* value <0.001 for both comparisons), with only minimal decline in sensitivity and NPV (*P* values 0.25 and 0.19 respectively).

### PBC Patients Have an Increased Serum IgG3

Twenty-two PBC patients were included as cholestatic liver disease controls. Serum IgG1, IgG2, and IgG4 and total IgG levels were within normal ranges. However, sIgG3 was markedly elevated in PBC, compared with PSC and IAC, patients (median values of 1.2, 0.4, and 0.4, respectively; *P* < 0.001, Kruskwal-Wallis' test; Table 1). Eleven (50%) PBC patients had a sIgG3 greater than the ULN.

## Discussion

The present study addresses the diagnostic dilemma of elevated serum IgG4 in differentiating patients with a chronic cholangiopathy otherwise compatible with PSC or IAC. Our data confirm that elevated serum IgG4 levels were found not only in IAC patients, but also in a considerable fraction (15%) of a large cohort of PSC patients, that the PPV of a sIgG4 in the lower elevated range between 1.4 and 2.8 g/L for IAC was only 28%, and that therefore serum IgG4 was insufficient to discriminate between PSC and IAC. Our data, for the first time, shows that the serum IgG4/IgG1 ratio proved to be helpful in distinguishing IAC from PSC in patients with moderately elevated serum IgG4.

In line with our findings, previous case series have shown elevated levels of IgG4 in 9%-27% of PSC patients.^19^–^23^,^27^ However, the number of included patients varied widely (34-285) and serum IgG1, IgG2, and IgG3 levels were never reported. In the current study, diagnostic accuracy provided by the sIgG4 only increased when increasing the sIgG4 cut-off value from 1.4 to 2.8 g/L (2× ULN), or even to 5.6 g/L (4× ULN), confirming findings of others.^27^,^28^ Particularly when sIgG4 is moderately elevated between 1.4 and 2.8 g/L (12% of PSC patients), the risk of misclassification of PSC as IAC and inadvertent corticosteroid treatment was evident.

IgG4-related sclerosing cholangitis can be demonstrated on liver needle biopsy and BD biopsy. Affected BDs characteristically show diffuse thickening with transmural sclerosing inflammation composed of a dense lymphoplasmacytic infiltrate and storiform pattern of fibrosis. IgG4 immunostaining of infiltrating plasmacytes may be observed diffusely. However, disease may be patchy and can be missed on a regular biopsy specimen, and histology should never be considered in isolation.^29^ The absolute IgG4 plasma count in a specimen cannot be used in isolation and must form part of an assessment considering the clinical picture, histological morphology, and the addition of an immunohistochemical (IHC) IgG4/IgG ratio (suggested at >40% to define IgG4-RD), as suggested by the consensus statement on the pathology of IgG4-RD.^11^ We feel that liver biopsy combined with cross-sectional imaging for other organ manifestations, particularly pancreatic disease, is important diagnostically in a subgroup with PSC and elevated IgG4, particularly because they seem to constitute a high-risk group.^19^ Histology is a crucial component to making an accurate diagnosis of IgG4-RD. Furthermore, whereas a biopsy carries a risk of complications in 5:1,000 people,^30^ steroids can also cause many serious adverse effects. Therefore, we feel that in patients with sclerosing cholangitis with equivocal results upon IgG spectrum measurement according to the proposed algorithm, liver histology should precede a trial of steroids.

In accord with previous studies, 23% of PSC patients in our study had hypergammaglobulinemia.^31^ Hypergammaglobulinemia may include elevation of different IgG subtypes and may mirror continuous nonspecific activation of the immune system. Whether elevated sIgG4 levels, in this context, are a cause or consequence of the severity of PSC remains elusive.

IAC patients showed, on average, an isolated elevation of IgG4, whereas, in PSC patients with an elevated IgG4, IgG1 was generally elevated as well, resulting in a lower IgG4/IgG1 ratio in PSC patients. Elevated sIgG4 levels in PSC have been associated with cirrhosis and a more severe disease course,^19^,^21^ but contamination of some PSC cohorts with undiagnosed IAC patients cannot be excluded—in one study several of the PSC patients with elevated IgG4 had a clinical profile suggestive of IgG4-RD, such as pancreatic involvement.^21^ Notably, cholangiocarcinoma (CCA) is associated with elevated sIgG4, especially in association with PSC, and patients can have elevated sIgG4 levels, typically between 1× and 4× ULN.^28^ Data analyzing other IgG subclasses in CCA are not available.

Our data can help in the interpretation of elevated serum IgG4 levels in the discrimination between IAC and PSC, but serum IgG subclass analyses do not represent 100% sensitive and specific tests. Therefore, the discrimination between IAC and PSC relies on more than serological measurements. Evaluating other diagnostic criteria for PSC or IAC, such as age and coexisting IBD, was beyond the scope of our study. PSC patients were not fully randomly selected from the two PSC cohorts, so bias with regard to age or coexisting IBD could not be excluded, and therefore we were unable to perform logistic regression for these factors. Nevertheless, in the present study, IAC patients were, on average, almost 20 years older at time of diagnosis than PSC patients (IAC; 62.5 years [SD, 14.1] vs. PSC; 44.0 years [SD, 16.2]). Even PSC patients with a sIgG4 >2.8 g/L were, on average, 13 years younger at time of diagnosis than IAC patients (data not shown). Furthermore, most IAC patients may, upon careful history taking, show to have various IgG4-RD organ manifestations. In addition, histopathological examination may help to differentiate between IgG4-RD and PSC.^9^

In the present study, we observed higher sIgG3 levels in PBC patients, compared to IAC and PSC patients. Antimitochondrial Abs (AMAs)—the diagnostic hallmark of PBC—are largely of IgG origin.^32^ AMAs are not restricted to one IgG subclass, but AMA-specific IgG3 titers are higher than IgG1 or IgG2 and associated with a more severe disease course.^33^,^34^

Compared to PSC patients, IAC patients had higher sIgG2 levels. We have no pathophysiological explanation for the difference in IgG2 levels between IAC and PSC. It appears possible that both IgG2 and IgG4 subtypes are induced in IAC upon long-standing antigen exposure. Notably, IgG2 and IgG4 are both induced in response to polysaccharide antigens, in contrast to IgG1 and IgG3.^35^ Another explanation could be that the measurement method is liable to false detection of slightly higher levels of IgG2 as a result of the great similarity of the IgG2 and IgG4 molecules.

There are several limitations to our study. Serum IgG subclasses of PSC and PBC patients were measured in frozen samples collected during the disease course instead of in fresh samples at time of diagnosis, as in IAC patients. We have done our utmost to ascertain the diagnosis in each patient; however, we did not perform additional IHC in all PSC patients for detection of IgG4^+^ plasma cells in PSC patients with an elevated sIgG4. However, none of the PSC patients had clinical signs of IgG4-RD (besides sclerosing cholangitis), which keeps the risk low that we misdiagnosed a PSC patient. Furthermore, CCA in the setting of PSC can induce elevated levels of IgG4 in up to 22% of patients.^28^ In the present study, 4 (1%) PSC patients in retrospect probably had an undiagnosed CCA at time of blood sampling; however, none had an elevated sIgG4.

In summary, we report on the first analysis of IgG subclasses in the largest PSC and IAC patient group to date, as well as in PBC controls. We confirm that sIgG4 was elevated in 15% of patients with an unchallenged PSC diagnosis. Our study demonstrates that serum IgG4 >1.4 g/L is not reliable enough to detect IAC in alleged PSC patients with a moderately elevated sIgG4 (>1.4 to <2.8 g/L). In this subgroup, incorporating the IgG4/IgG1 ratio with a cutoff at 0.24 in the diagnostic algorithm significantly improves PPV and specificity and is therefore helpful in distinguishing IAC from PSC. External validation of our findings is now warranted.

## Abbreviations

Abs: antibodies
AIP: autoimmune pancreatitis
ALP: alkaline phosphatase
AMA: antimitochondrial Ab
AUROC: area under the ROC curve
BDs: bile ducts
CCA: cholangiocarcinoma
CI: confidence interval
ERC: endoscopic retrograde cholangiography
HPF: high-power field
IAC: IgG4-associated cholangitis
IBD: inflammatory bowel disease
Ig: immunoglobulin
IgG4-RD: IgG4-related disease
IHC: immunohistochemistry
IRSC: IgG4-related sclerosing cholangitis
IQR: interquartile range
LT: liver transplantation
MRC: magnetic resonance cholangiography
NL: the Netherlands
NPV: negative predictive value
PBC: primary biliary cirrhosis
PPV: positive predictive value
PSC: primary sclerosing cholangitis
ROC: receiver operator characteristic
SD: standard deviation
SSC: secondary sclerosing cholangitis
sIg: specific Ig
UDCA: ursodeoxycholic acid
UK: United Kingdom
ULN: upper limit of normal
