# Supplementary material for: Serum Immunoglobulin G4 and Immunoglobulin G1 for Distinguishing Immunoglobulin G4-Associated Cholangitis From Primary Sclerosing Cholangitis
Source: Hepatology. 2014 Apr 1;59(5):1954–63. doi: 10.1002/hep.26977 (PMC4489327; doi:10.1002/hep.26977)
Supplement: Supplementary file 2 — Supplementary Information Table 1. [file hep0059-1954-sd2.doc]

**Supplementary Table 1.** Number of biopsies evaluated and percentage of IgG4 positive stainings for PSC patients with a serum IgG4 > 1.4 g/L and IAC patients with a serum IgG4 <1.4 g/L.

|  | **PSC** |  |  | **IAC** |  |  |
| --- | --- | --- | --- | --- | --- | --- |
|  | **IgG4 > 1,4 g/L** |  |  | **IgG4 < 1,4 g/L** |  |  |
|  | **No. (%)** | **classical histology of IAC (2 or more)** | **tissue IgG4 high (>10/HPF biopsy; >50/HPF resection)** | **No. (%)** | **classical histology of IAC (2 or more)** | **tissue IgG4 high (>10/HPF biopsy; >50/HPF resection)** |
| **any histology** | 29/45 (64) | 0/29 | 7/16 | 5/7 (71) | 4/5 | 3/5 |
| **any biopsy** | 29/45 (64) | 0/29 | 7/16 | 5/7 (71) | 4/5 | 3/5 |
| liver | 29 | 0/29 | 3/16 | 0 | 0 | 0 |
| bile duct | 0 | 0 | 0 | 0 | 0 | 0 |
| pancreas | 0 | 0 | 0 | 3/7 | 3/3 | 2/3 |
| other | 22 (colon) | 0 | 7/16 | 2/7 (ampulla, renal) | 1/2 | 1/2 |
| **any resection** | 1/45 (2) | 0/45 | 0/45 | 3/7 (43) | 3/3 | 3/3 |
| liver | 1 | 0 | 0 | 1 | 1/1 | 1/1 |
| bile duct | 0 | 0 | 0 | 1 | 1/1 | 1/1 |
| pancreas | 0 | 0 | 0 | 2 | 2/2 | 2/2 |
| other | 6 (colon) | 0 | unknown | 0 | 0 | 0 |
